# Liberating the Postpartum Body: The Effects of Movement on Body Image Amongst Postpartum Ultra-Orthodox Women in Israel

**DOI:** 10.1007/s10943-024-02160-y

**Published:** 2024-11-05

**Authors:** Gabrielle Fine, Einat Shuper Engelhard

**Affiliations:** 1 School of Creative Art Therapies, Faculty of Social Welfare & Health Sciences, University of Haifa, Haifa, Israel; 2 Dance Movement Therapy program, Graduate School of Creative Art Therapies. Faculty of Social Welfare & Health Sciences, Emili Sagol Creative Arts Therapies Research Center, University of Haifa, Mt. Carmel, 31905 Haifa, Israel; 3 Graduate School of Creative Art Therapies. Faculty of Humanities & Social Sciences, Kibbutzim College of Education, 149 Derech Namir, 62507 Tel Aviv, Israel

**Keywords:** Postpartum, Religion, Body image, Mixed-method Research, Ultra-Orthodox women

## Abstract

Body image is a major postpartum problem negatively correlated with postpartum depression. The present study tests this correlation amongst ultra-Orthodox women in Israel and analyses whether movement classes are effective in improving postpartum body image. Utilising a mixed-methods approach, the study followed two groups of ultra-Orthodox postpartum women (*n* = 45): one group that participated in movement classes (*n* = 23) and a comparison group that did not (*n* = 22). Results of the study found a negative correlation between depression and body image amongst ultra-Orthodox women (*r*(53) =  −0.342, *p* < .05) and a trend of movement classes positively affecting postpartum body image (*t*(43) = 1.388, *p* = .086). Furthermore, semi-structured interviews found three themes as to how movement 1. Regulates emotions, 2. Releases stress and 3. Places the mother at the centre. Discussion of the results highlights the importance of maintaining positive body image and emotional health in the postpartum period, and that, according to the perception of participants in this study, movement positively contributed to their sense of self and coping abilities. These connections are critical regarding ultra-Orthodox women, whose birth rate is significantly higher relative to broader society. The current study should serve as a basis for encouraging postpartum movement participation amongst ultra-Orthodox and other similar conservative religious sectors of society.

## Introduction

The postpartum period can be filled with joy, satisfaction, and elation while, at the same time, fraught with unrealistic expectations of being the perfect mother and finding the balance between caring for a newborn child and caring for oneself (Lambermon et al., 2020). Body image problems are common during the postpartum period, with women adapting to their new postpartum and post-delivery weight and bodies, as well as altered self-image and high levels of fatigue (Clark et al., 2009; Walker et al., 2002). Body image is described as one's feelings, emotions, attitude, and behaviour toward one’s body (Cash, 2012). Negative postpartum body image or body dissatisfaction has been linked to emotional distress (Hartley et al., 2018) as well as to postpartum depression (Clark et al., 2009; Downs et al., 2008; Green et al., 2006; Rallis et al., 2007; Sweeney & Fingerhut, 2013). Negative body image has also been found to predict the likelihood of developing postpartum depression (Roomruangwong et al., 2017; Walker et al., 2002). Whether causal or correlative, the postpartum period is one ‘during which physical changes are innately linked to perceptions and feelings about the body self’ (Walker et al., 2002).

Movement has been found to have a positive effect on postpartum depression levels, with prenatal yoga reducing postpartum depression symptoms (Bershadsky et al., 2014), and various other movement interventions which were found to have a positive effect on lowering depression scores amongst postpartum women (Daley et al., 2015; Norman et al., 2010). Movement has been found to positively affect body image amongst many age groups (Așçı, 2002; Campbell & Hausenblas, 2009; Duncan et al., 2009; Hausenblas & Fallon, 2006; Marconcin et al., 2021; Sabiston et al., 2019; Vocks et al., 2009). However, research into these connections in the postpartum period is scant.

A study examining the connections between exercise, body image, and depression in the period before and after childbirth, found that the three elements were associated among pre and postpartum time points. Results also revealed that exercise had a moderating influence on body image and depression in early pregnancy, as well as higher body image satisfaction in the third trimester (Downs et al., 2008). An additional study that examined factors influencing body image during the postpartum period found a significant negative correlation between body image and pre-pregnancy weight, weight gain during pregnancy, breastfeeding duration, and the number of births. Exercise was also found to significantly impact body image, albeit limited relative to age and number of births (Erbil et al., 2012). Thus, the basic connections are there and need further research and addressing.

The postpartum movement-body image connection is critical given the postpartum-specific body image issues mothers deal with and the negative effects these can have on the mother, the mother–child relationship, and the eventual postpartum complications negative body image is correlated with.

Body image is also affected by social, cultural, and religious elements (Chaker et al., 2015; Inman et al., 2023; Tiggemann & Hage, 2019). The body image construct within the religious world can have different criteria entirely, even more so in the ultra-Orthodox sector, a group that adheres to a strict legal framework (*Halakha*) (Prins-Engelsman & Cwikel, 2023). These laws govern every aspect of ultra-Orthodox life and put a strong emphasis on female modesty, with an aim of setting them apart from secular sectors of society.

Ultra-Orthodox Jews adhere to the most extreme forms of modesty within Orthodox Jewry, which encompasses a spectrum ranging from Modern Orthodox, who often adopt a more liberal approach (Seigelshifer & Hartman, 2011), to the highly conservative Ultra-Orthodox communities. The ultra-Orthodox concept of modesty includes rules regulating women’s behaviour, appearance, and conduct. Ultra-Orthodox women avoid revealing clothing or attire that accentuates their figure, cover all of their hair after marriage, and are prohibited from singing or dancing in the presence of men (Bachner-Melman & Zohar, 2019; Yafeh, 2007).

This religious framework significantly influences women’s perceptions of their bodies and its purpose (Geller et al., 2023; Suskin & Al-Yagon, 2020), and governs its use (Stadler, 2009; Yafeh, 2007). This includes an unwavering ultra-Orthodox commitment to the commandment to procreate (Teman & Ivry, 2021). Consequently, women in the ultra-Orthodox sector have a higher birth rate relative to other Jewish women in Israel, with an average of 6.4 children as opposed to 2.5 (Cahaner & Malach, 2023). This demographic reality places ultra-Orthodox women in a uniquely body image-sensitive position. Furthermore, short inter-pregnancy intervals are common in this community, which has been found to statistically affect mothers’ postpartum moods (Gurel & Gurel, 2000) and their physical bodies (Conde-Agudelo, et al., 2006).

Limited research exists on movement and body image among ultra-Orthodox women. The little research that does exist found that movement positively affects various body image measures within this unique sector of society (Suskin, 2018). However, these connections have yet to be explored specifically in the postpartum period for ultra-Orthodox women, presenting a significant gap in the literature.

## Purpose

The present study, therefore had four major aims:

1. To analyse whether body image is correlated with depression in the postpartum period amongst ultra-Orthodox religious women.
2. To analyse the connection between depression levels and women who chose to participate in movement classes, and those who chose not to participate in classes.
3. To analyse the effects of movement on body image among ultra-Orthodox women in the postpartum period.
4. To understand the psychological elements involved in the movement experience for postpartum ultra-Orthodox women.

## Method

### Research Design

The present study used an explanatory sequential mixed methods design (Creswell, 2014). First, the quantitative element was longitudinal research, using a quasi-experimental non-equivalent (pre-test and post-test) control group design. It was used to test the first three of the four aims. The use of a qualitative paradigm allows the researcher to study a phenomenon through the words, perspectives, feelings, and beliefs conveyed by participants, and in doing so, allows the researcher to gain insight into the participants’ subjective experience (Shkedi, 2003). In qualitative research, the researcher's background is of ethical importance (Creswell, 2014), and self-reflecting processes were implemented during the interview process to limit the researcher’s bias. The researcher who conducted the interview’s connection to this subject is that she is a mother and an orthodox Jew, though does not identify herself as belonging to the ultra-Orthodox sector.

## Participants

The study included 45 postpartum ultra-Orthodox women. Participants were recruited using a combination of snowball sampling via community representatives, and convenience sampling using flyers and posters placed in studios, gyms, and dance schools located in ultra-Orthodox neighbourhoods.

For the movement group, inclusion criteria required that participants be ultra-Orthodox (by self-definition), be within six months postpartum, have chosen to participate in movement classes unrelated to the study, and be within a week of starting movement classes after giving birth. A total of 23 women met these criteria and were included in the movement group. They participated in at least one hour of movement classes, once a week, for twelve weeks.

The comparison group consisted of 22 women who were also ultra-Orthodox (by self-definition) and were within six months postpartum, but who had expressed a choice not to participate in movement classes.

Participants ranged in age from 20 to 41 years, with an average of three months postpartum at the time of joining the study and an average of four children. Some participants chose to further identify themselves as belonging to specific ultra-Orthodox subcategories, including Hassidic (*n* = 5), Habad (*n* = 8), Lithuanian (*n* = 2), and Sephardi (*n *= 2). No significant statistical differences were found between the movement and comparison groups (further information can be found in Table 2).

Participants in the movement group attended dance or Pilates classes, two types of classes that include both cardiovascular endurance and muscular strength. Participants in the movement group cited a variety of reasons for attending classes. Six participants indicated that their reason for participating was for their mental health, while 14 participants cited physical health. Two participants indicated that it was for their external appearance, and one participant did not provide an answer.

In addition to the 45 participants who completed the full research process, five began participating in the movement group but did not consistently attend classes. An additional five participants in the comparison group chose not to complete all the questionnaires and dropped out after the first round of questionnaires (*n* = 3), or after the second (*n* = 2).

The qualitative section of the research included seven participants, at which point saturation had been reached. The participants were recruited from the movement group in the quantitative section of this research and were subsequently invited to participate in the qualitative section, to which they agreed. Five of the participants identified themselves as ultra-Orthodox without any subcategory, one identified herself as Hassidic, specifically from the Belz sector, and one as Sephardi.

The research was approved by the ethics committee at Haifa University, approval number 3059.

## Research Procedure

Interested participants were given the researcher’s details, at which point an introductory phone call was held between the researcher and the participants. During this call, the researcher explained the aim of the research and all stages of the study to the potential participants. A consent form was read, and verbal approval was given and recorded. At the first stage (T = 0), participants were asked to answer three questionnaires. First, a socio-demographic questionnaire, which included questions about the participant’s age, family, socio-economic background, movement participation, and religious affiliation.

Second, participants answered the Edinburgh Postnatal Depression Scale (EPDS). The Hebrew translation by Glasser and Barell (1999) of the original questionnaire by Cox et al., (1987) was used. A study by Prins-Engelsman and Cwikel (2023) which utilised the Hebrew questionnaire, reported Cronbach *α* = 0.86. The Cronbach *α* = 0.87 for the original English questionnaire (Cox et al., 1987). The scale comprises 10 questions and addresses subjects including happiness (“I have been so unhappy that I have been crying”) and anxiety (“I have been anxious or worried for no good reason”). Participants responded on a 4-point Likert scale, with answers ranging from zero to three; the overall score of the questionnaire is the sum of the items in it.

Third and lastly for this first stage, participants were asked to complete the Body Appreciation Scale. The Hebrew translation by Ben David (2009) of the original questionnaire by Avalos et al. (2005) was used. In order to make it culturally relevant for ultra-Orthodox participants, a question regarding whether seeing women in the media is connected to forming one’s opinion on body image, was replaced with how seeing women in society affects body image, as per Suskin’s adaptation of the questionnaire for ultra-Orthodox participants (Suskin, 2018). The scale comprises 13 questions and addresses subjects including respecting one’s body, (“I respect my body”), feelings towards one’s body (“my feelings towards my body are positive, for the most part”), and attentiveness to one’s bodily needs (“I am attentive to my body’s needs”). Participants respond on a 5-point Likert scale, ranging from 1 (never) to 5 (always). A prior study by Avalos et al. (2005) reported Cronbach *α* = 0.94. The Cronbach *α* = 0.88 for Suskin’s research after adapting it to the ultra-Orthodox participants (Suskin, 2018).

Many ultra-Orthodox families do not use smartphones or have internet access; therefore the researcher called the participants to answer the questionnaires over the phone. In a subsequent phone call six weeks later (T = 1), participants again answered the Body Appreciation Scale, and then again six weeks after that (T = 2).

Seven of the participants in the movement group subsequently agreed to participate in semi-structured interviews. The same researcher conducted all interviews. The researcher offered participants to do the interviews in their homes or in a studio, and emphasised that if they were at home, at a time where participants would have privacy and be able to talk freely. Most opted to do the interview at home (*n* = 3) when children were asleep and their spouses were not home. Some participants opted to do the interviews in a studio (*n* = 2) when there were no classes taking place and the researcher and participants had full privacy, and some preferred by phone (*n* = 2). Interviews lasted approximately one hour.

## Statistical Analysis

### Quantitative

For continuous variables, means and standard deviations were calculated, and frequencies and percentages were calculated for all categorical variables. As is the most appropriate form of analysis for a study with 2 groups and 3 measures, a repeated ANOVA was conducted to test the differences between the movement group and the non-movement group and their body image. Due to the sample size in the current study, the statistical power (1-*β*) was insufficient. Consequently, the most reliable method of analysis was to compare the differences in deltas between the measures. Two t-tests were conducted comparing the delta (T1—T0) in body image between the groups and the delta (T2-T0) between the groups. The criterion for significance of Alpha (*α*) = 0.05 (two-sided). To test the correlation between depression levels and body image, a Pearson correlation was conducted. Statistical analyses were conducted using the SPSS statistical software (Version 21).

### Qualitative

Thematic analysis of the semi-structured interviews used an inductive approach. Interviews were recorded and transcribed, and answers were analysed as follows: 1. Data organisation: All relevant data was transcribed from the recordings. 2. Reading the material: All the data was read and general ideas that arose were identified. 3. Coding: Data was organised and categorised. 4. Description: Coding was used to ascertain themes which were the major findings of the study. 5. Connecting the themes: A tree diagram, or category tree, was used. On the horizontal plane were the central/basic categories, and on the vertical plane were sub-categories relating to the family of the basic categories listed above. 6. Data interpretation: what was learned from the research, how this related to previous research and future research direction (Creswell, 2014). Results were reached through back-and-forth examination between the researchers, and in-depth discussion of the themes and sub-themes until an agreement was reached.

## Results

### Quantitative

As shown in Table 1, a statistically significant negative correlation was found between depression levels (EPDS) and body image scores at T_0,_ *r*(53) =  −0.342, *p* = 0.011. As shown in Table 2, no significant differences were found between the groups in terms of age, number of children, interval from birth to first measurement, number of years of schooling, rate of employed women, or depression levels. A 3 (time) X 2 (groups) repeated ANOVA was conducted to test the differences between the movement group and non-movement group and their body image. Results of the ANOVA test are shown in Table 3, with no significant differences found between the groups *F*(2,86) = 1.495, *p* = 0.230.

**Table 1:** Correlation between depression levels and body image amongst postpartum ultra-Orthodox women

	VAR00001	VAR00002
VAR00001 Pearson correlation	1	−342^*^
Sig.(2-tailed)		.011
N	55	55
VAR00002 Pearson correlation	−342^*^	1
Sig.(2-tailed)	.011	
N	55	55

**Table 2:** Demographic characteristics and ruling out differences between groups

Variable | Movement	Yes (*n* = 23)	No (*n* = 22)	*P*-value
Age	29.13 (5.19)	30.39 (5.43)	.432
Number of children	4.00 (2.26)	4.23 (2.51)	.751
Interval (birth—first measure)	3.13 (1.32)	3.23 (1.77)	.837
Years of schooling	14.83 (1.59)	14.09 (2.02)	.181
Depression (EPDS)	5.74 (3.83)	4.18 (3.63)	.169
Employed, n (%)	13 (57)	10 (44)	.172

**Table 3:** Results of the repeated ANOVA test showing the difference between groups in body image across measurements

Movement| Measure	First	Second	Third	*P*-value
Yes	3.91 (0.54)	4.06 (0.47)	4.14 (0.50)	.230
No	3.98 (0.59)	3.98 (0.58)	4.05 (0.57)	

Table 4 shows the subsequent results of the two t-tests comparing the groups: the delta of each group T1—T0 and the delta of each group T2-T0. A statistical trend was found when comparing the differences in the deltas between the groups. In the movement group, a mean delta of 0.23 (± 0.45) was found, while a mean delta of 0.07 (± 0.31) was found in the non-movement group, *t*(43) = 1.423, *p* = 0.081. The difference in the delta between each group’s first and second measures also revealed a trend of the difference between the groups, *t*(43) = 1.388, *p* = 0.086.

**Table 4:** Results of t-tests comparing the groups: the delta of each group T1–T0 and the delta of each group T2-T0

Movement | Measure	First- Second	*P*-value	First-Third	*P*-value
Yes	0.15 (0.30)	.086	0.23 (0.45)	.081
No	0.01 (0.39)		0.07 (0.31)	

## Qualitative

Given that the quantitative side of the research found a trend rather than a statistically significant result, seven of the ultra-Orthodox participants were approached and agreed to be interviewed. The interviews were conducted to further understand the psychological and emotional elements underpinning the changes in body image scores found amongst participants enrolled in movement classes. The demographic characteristics of participants are shown in Table 5.

**Table 5:** Demographic characteristics for qualitative data set

	Mean (SD)
Age	26.55 (3.91)
Number of Children	2.86 (1.57)
Years of schooling	14.86 (1.77)
Months post birth	4.99 (0.98)

Through thematic analysis, movement was found to serve as a coping tool in the postpartum period on three levels, as shown in Table 6. First, 1. movement regulates negative emotions and evokes positive emotions. It a. elevates feelings of happiness and optimism, b. silences negative inner thoughts, c. improves relationships at home, and d. helps to relax. Second, 2. movement as an outlet and release from stress, as a. an outlet for feelings or emotions, and b. as a release. Finally, the mothers felt that 3. movement places the mother at the centre, and creates a. a place just for them, b. balances dependence in the dyad relationship, and creates a sense of c. self-worth in relation to others.

**Table 6:** Movement as a coping tool in the postpartum period

(I) Movement regulates negative emotions and evokes positive emotions	(2) Movement as an outlet and release from stress	(3) Movement places the mother at the centre
(a) Elevates feelings of happiness and optimism	(a) An outlet for feelings or emotions	(a) A place just for them
(b) Silences negative inner thoughts	(b) As a release	(b) Balances dependence in the dyad relationship
(c) Improves relationships at home		(c) Self-worth in relation to others
(d) Helps to relax		

### Movement Regulates Negative Emotions and Evokes Positive Emotions

#### Elevates Feelings of Happiness and Optimism

Nearly all mothers (*n* = 6) stated that movement stimulated positive feelings, with an emphasis on feelings of joy, happiness, and optimism. As one mother related: “For me, it [dancing] evokes a lot of joy… That’s it, it truly makes me very, very happy” (interview 7, lines 130–132). Mothers also shared that movement provided an opportunity for them to change their mood in a more positive direction and that “it definitely puts me in a better mood. Like tonight, once I put my child to sleep, I was in a blah mood, no patience for anything, and then I made up my mind that I am coming, and now I am in the best mood ever” (interview 5, lines 16–18). In contrast, one mother described her movement experience as one that “arouses frustration and disappointment”, (interview 4, line 112) in light of having felt that her movement abilities were poorer than those of the rest of the group.

#### Silences Negative Inner Thoughts

Movement enabled more than a third of the mothers (*n* = 3) to silence thoughts of lack of self-confidence or insecurity about their abilities: “It silences thoughts like I can’t, I can’t!” (interview 4, line 238). Mothers also reported that movement helped them quell feelings of failure, whether regarding their personal shortcomings, body image, or relationships with their children in the postpartum period. They felt that movement helped them accept the difficulties, complexities, and impossibility of achieving perfection during this early stage of parenting. One mother’s observation is particularly revealing: “There may be things that we do or don’t succeed, it is all OK. I can carry on trying. This is true about movement, but it’s also sort of a message for life” (interview 3, lines 130–132).

#### Improves Relationships at Home

Most of the mothers (*n* = 5) shared how the movement experience influenced their lives outside the studio and enhanced their home environment by bringing movement into the home and into the lives of their children. In one mother’s opinion: “I think that a house with a lot of movement is a house with a lot of happiness” (interview 1, line 141). In addition, they felt that movement improved their moods and enabled them to be more loving and patient towards their children: “I go home to my family, and I just want to eat them up” (interview 2, lines 208–209).

#### Helps to Relax

More than half of the mothers (*n* = 4) described how movement helped them to relax, and through that, achieve more inner peace of mind. As one mother explained: “You are familiar with…having a sort of relaxation during movement… it’s like something for the soul” (interview 2, lines 207–208). In addition, mothers described this experience of relaxation as something new or surprising, even unfamiliar: “I know how to relax, I discovered it, I have that ability” (interview 4, line 241).

## Movement as an Outlet and Release from Stress

### An Outlet for Feelings or Emotions

More than half of the mothers (*n* = 4) said that movement is a time and avenue for them to actively express their emotions, “I release my emotions through exercise” (interview 6, line 22). Mothers described the importance of expressing emotions through movement and the negative consequences if they do not do it, “If I do not release enough energy, it really accumulates a lot of nerves and anger, and it is a shame” (interview 1, lines 103–104). When they succeeded in unleashing their emotions through movement, mothers described themselves as feeling more energised, and invigorated.

### As a Release

Nearly all mothers (*n* = 6) described their movement experience as a release or liberating and described this release in different ways. One mother spoke of movement as liberating her, “It really liberates me” (interview 2, line 87) or movement as releasing tension. For many mothers, release was synonymous with the word “fun”; “It is always an enjoyable and liberating feeling” (interview 6, line 31). One mother emphasised the importance of the concept of fun as a return to a period in her life when she felt younger and freer, “At some point in life, it often dissolves, and we do not do things that are fun for us anymore” (interview 6, lines 37–38). Mothers also described the liberation or release experienced as “freedom,” “important,” and the “the best feeling in the world.”

## Movement Places the Mother at the Centre

### A Place Just for them

Nearly all mothers (*n* = 6) shared that movement provided them with a time, space, and unique experience of an activity solely for themselves. One mother excitedly said, “I feel like I am doing something that is just for me, and it is fun just for me. And I chose it” (interview 3, lines 27–28). Mothers talked about how doing something just for themselves is rare in the early years of motherhood and that “there is much less awareness of putting yourself somewhat first” (interview 3, lines 162–163). They also emphasised both the difficulty and importance of getting out of the usual routine and taking a moment for themselves, and the satisfaction that comes when they manage to get to a class and do something for themselves, with one mother expressing triumphantly “such satisfaction from doing something that is truly mine” (interview 3, lines 28–29).

### Balances Dependence in the Dyad Relationship

Most of the mothers (*n* = 5) described the pressure they feel of their children and household being dependent on them, and how through movement, they learned how to leave this dependence outside the door, to forget the pressure for a moment, and focus solely on themselves. As one mother said, “I am leaving now. I left my husband with the kids. They went out. I said (to myself), it is okay, they will calm down. The important thing is that I made a decision, I have to go out” (interview 3, lines 102–103). On a very physical level, mothers (*n* = 2) described the difficulty and physical pressure they feel of a baby dependent on their physical body, either through breastfeeding or everyday life, and how movement balances this dependence, as one mother said, “You carry a 5 kg baby all the time, and other children you need to lift sometimes—so I feel that only through exercise, I release myself, and I regain some stability.”(interview 2, lines 107–109).

### Self-worth in Relation to Others

The movement experience brought to the fore a sense of competitiveness among more than half of the mothers (*n* = 4), generating feelings of higher self-esteem compared to their peer group. Mothers described themselves as better than others in the group, as slimmer, with higher energy levels, more satisfied, and more capable, as one mother expressed proudly, “I like to dance and work hard, I was one of the better ones” (interview 1, line 54). The movement experience also allowed mothers to feel personally superior to others, with one mother commenting, “Definitely compared to friends who do not exercise, I have more energy” (interview 5, lines 72–73). However, two mothers talked about their self-esteem being lowered when comparing themselves to other mothers in their movement group, leaving them feeling incapable or inferior, “I look at other women who do the exercises really well and easily, and I say: I wish I could get to that level” (interview 4, lines 109–110).

## Discussion

A negative correlation was found between depression levels and body image, in line with previous research (Roomruangwong et al., 2017; Walker et al., 2002), with the current research adding that this correlation is also relevant to ultra-Orthodox women. Additionally, there were no connections found between women who chose to participate in movement and depression levels. Hence, depression was not a factor in explaining why women chose to enrol in movement classes or not.

Findings regarding the hypothesis that women involved in movement will show a greater improvement in body image scores were inconclusive. However, a trend emerged of movement positively affecting body image in the postpartum period, which is consistent with previous research on the positive effects of movement on body image (Așçı, 2002; Duncan et al., 2009; Vocks et al., 2009). In contrast, the results in the current research were inconclusive. This could be attributed to this study’s limited sample size—45 participants, compared to a range of 65 to 138 participants in the studies cited.

It is also possible that the frequency of the movement intervention may have affected the strength of the results. In the current study, women participated in movement classes once a week for 12 weeks, compared to a range of higher frequency of weekly classes, or more hours of classes per week, in previously mentioned studies. This infrequency may have reduced its effect, as suggested in a meta-analysis which found that the frequency of movement (exercise) per week moderated the level of their effect on body image scores (Campbell & Hausenblas, 2009).

In the present study, the average timing of participants starting the research for both groups was slightly more than three months postpartum, with women finishing their intervention three months later. Previous research is divided as to how long after birth mothers are prone to experiencing body image issues (Solorzano et al., 2022), with findings ranging from three months postpartum (Green et al., 2006) through six weeks to six months (Clark et al., 2009; Rallis et al., 2007), and zero to nine months (Gjerdingen et al., 2009). Given this range and the timing of our study, it appears that participants in the current study were involved in movement classes at the height of potential postpartum body image complications. While movement did have a positive effect, it is possible this effect was slower or perhaps more difficult to achieve due to the timing.

The uniqueness of the ultra-Orthodox society may also shed light on understanding the results of this study. Ultra-Orthodox women are often the family’s breadwinners. This is in order to support their husbands who are engaged in full-time Torah study in *Yeshivot*, Jewish seminaries, where ultra-Orthodox men devote themselves and their time (Frenkel, 2018; Kook & Harel-Shalev, 2020; Shahar, 2015). In addition, ultra-Orthodox women are primarily responsible for running the household (Caplan, 2003; Gado et al., 2023; Ilan, 2000) and raising their children, all while having a higher than average number of children (participants in this study had an average of four children under the age of seven to care for). This all-encompassing role leads to higher levels of fatigue and elevated levels of stress, which has been found to negatively affect body image (Marcotte et al., 2002; Murray et al., 2011; Tylka & Wood-Barcalow, 2015), perhaps reducing the effects of movement on body image in the current research.

## The Subjective Experience of Women in Movement

Through qualitative semi-structured interviews, the present study found that the movement experience was perceived by mothers as promoting positive change in self-esteem, and individual well-being during the postpartum period. Movement classes evoked positive emotional experiences, such as feelings of happiness, joy, and capability. Therapeutic elements arose from the movement experience, such as an outlet for emotions, a release, or a way for mothers to regulate their emotions, anger, or frustration, consistent with previous research findings (Shafir et al., 2013).

This study contributes to the existing and growing research into the field of movement as a means for emotional and psychological change (Zaides et al., 2021; Elboim-Gabyzon et al., 2022; Doonan & Bräuninger, 2015; Ortuño-Ibarra & Rodríguez-Jiménez, 2022; Streater, 2022). However, body-centred approaches present an ideological complexity within the ultra-Orthodox world, where the body is traditionally perceived as secondary, a vessel for elevating the soul, with that hierarchy conscientiously in place (Hakak, 2009; Suskin & Al-Yagon, 2020). Findings in the current study emphasise that despite these cultural and religious perspectives, movement and the body were a powerful source of coping and postpartum well-being for these women, and therefore, should not be overlooked in postpartum care for this population.

Finally, women in this study highlighted that movement enabled them to place themselves at the centre, to relax and cope, aligning with research into postpartum self-connection and self-care as essential for new mothers’ well-being (Lambermon et al., 2020; Rose et al., 2024). This is particularly significant in ultra-Orthodox society, given their high birth rate and where women's numerous roles and cultural norms often limit self-care opportunities (Leiter et al., 2019). Despite cultural and postpartum pressures, findings in the current research showed that movement successfully provided these mothers with a crucial opportunity to develop their sense of self. These findings extend previous research on movement’s role in promoting well-being and individuality among ultra-Orthodox women (Suskin, 2018) and demonstrate its relevance to the postpartum period. Additionally, the results align with self-reported self-care benefits of postpartum movement (Horton & Robledo, 2024), highlighting their applicability to ultra-Orthodox society.

This study therefore bridges two important areas of research, movement for body image and postpartum well-being, and in this unique cultural context. While acknowledging culturally specific themes to ultra-Orthodox society, these findings provide a firm foundation for the practical promotion of postpartum movement classes amongst ultra-Orthodox women. However, the implementation should be culturally sensitive and relevant to maximise participation in this demographic.

## Limitations and Future Research

The current research was focussed on ultra-Orthodox women, who have limited exposure to technology, including many not having internet or smartphones. Therefore, questionnaires were conducted for all participants by phone, which could potentially limit the participant’s ability to answer freely and openly, due to their wanting to paint themselves as more socially desirable (Holbrook et al., 2003). An additional limitation is the non-randomised allocation of participants. Due to the hesitancy of this sector of society to engage in movement classes, and caution towards academia as a whole, in order to gain access to this relatively un-accessed sector of society, the research followed participants who were enrolled in ultra-Orthodox approved classes. Furthermore, due to the busy lifestyle of the participants, two of them preferred to be interviewed by phone, which is less desirable for research purposes. Additionally, movement was categorised as dance or Pilates, two different types of movement classes with different qualities and intensities of movement. Finally, while the current study explored the reasons why participants chose to participate in movement classes, it did not explore the reasons why ultra-Orthodox postpartum women chose not to participate, a topic that needs further exploration.

Future research should expand upon and differentiate between these types of classes and broaden the research to include other types of movement classes. Future research should also expand the number of participants, the number of classes and the intensity of classes per week to determine if the effects on body image could be larger or more conclusive. Finally, future research should explore prior exposure to and participation in movement classes, as well as knowledge about postpartum challenges, particularly postpartum depression.

## Conclusion

Despite these limitations, the present study contributed to a deeper understanding of healthy postpartum care and the interplay between religion, body image, and depression during the postpartum period. Maintaining a psychologically and emotionally positive approach towards one’s body during this time is both extremely difficult, but extremely important, given correlations found between negative body image and postpartum depression, and the ramifications that can have on the mothers’ emotional welfare (Hartley et al., 2018), and on the family as a whole. This is ever relevant for ultra-Orthodox women, with their higher-than-average birth rate and many body-specific regulations to follow.

Furthermore, movement in the postpartum period was found to positively contribute to ultra-Orthodox women’s mental health. Movement was found to be an important coping tool for women, positively contributing to their emotional well-being, to their body image, to their development of self, and to their ability to regulate their emotions during this time. Continued research in this field should focus on how to further offer movement in a culturally sensitive and relevant way to women in the ultra-Orthodox sector and other conservative religious sectors in order to further support postpartum participation.
